# Biotechnological approaches and therapeutic potential of mitochondria transfer and transplantation

**DOI:** 10.1038/s41467-025-61239-6

**Published:** 2025-07-01

**Authors:** Gokhan Burcin Kubat, Pasquale Picone, Erkan Tuncay, Leila Aryan, Antonella Girgenti, Laura Palumbo, Ibrahim Turkel, Firat Akat, Keshav K. Singh, Domenico Nuzzo

**Affiliations:** 1 Institute of Transplantation and Gene Sciences, Baskent University, Ankara, Türkiye; 2 Department of Exercise and Sport Sciences, Faculty of Health Sciences, Baskent University, Ankara, Türkiye; 3 Consiglio Nazionale delle Ricerche, Istituto per la Ricerca e l’Innovazione Biomedica, Palermo, Italy; 4 Department of Biophysics, Faculty of Medicine, Ankara University, Ankara, Türkiye; 5 Department of Interdisciplinary Neuroscience, Ankara University, Ankara, Türkiye; 6 Department of Exercise and Sport Sciences, Faculty of Sport Sciences, Hacettepe University, Ankara, Türkiye; 7 Department of Physiology, Faculty of Medicine, Ankara University, Ankara, Türkiye; 8 Departments of Genetics, Dermatology and Pathology, Heersink School of Medicine, University of Alabama at Birmingham, Birmingham, AL USA

**Keywords:** Biotechnology, Molecular medicine, Regenerative medicine, Nanobiotechnology

## Abstract

Mitochondrial dysfunction contributes to aging and diseases like neurodegeneration and cardiovascular disorders. Mitochondria transfer and transplantation (MTT) represent promising therapeutic strategies aimed at restoring cellular function by introducing functional mitochondria into damaged cells. However, challenges like transfer efficiency, stability, and cellular integration limit clinical application. Recent biotechnological advances—such as liposomes, extracellular vesicles, and surface modifications—enhance mitochondrial protection, targeting, and biocompatibility. This Perspective highlights recent progress in MTT, its therapeutic potential, and current limitations. We also discuss the need for scalable, clinically translatable approaches and appropriate regulatory frameworks to realize the full potential of mitochondria-based nanotherapies in modern medicine.

## Abstract (web summary)

Mitochondrial dysfunction is involved in several disease states, mitochondria transfer and transplantation have the potential to address this. Here, the authors review recent advancements and challenges with emphasis on the contribution and potential of biotechnology in mitochondria transfer and transplantation.

## Introduction

Mitochondria play a critical role in energy production, regulation of apoptosis, and maintenance of cellular homeostasis^1^. Mitochondrial dysfunction refers to the organelle’s inability to effectively carry out its essential functions, including adenosine triphosphate (ATP) production, mitochondrial biogenesis, ionic homeostasis at both intracellular and organelle levels, and regulation of cell fate^2–6^. The diagnosis of mitochondrial dysfunction requires an integrated assessment of organelle structure and function, alongside the availability of metabolic substrates and reducing equivalents^7^. Isolated mitochondria have been identified as materials that can correlate with various cellular parameters such as oxidative stress levels, apoptotic signaling, and bioenergetics capacity^7–10^. A multitude of studies has demonstrated a strong correlation between mitochondrial dysfunction and a wide range of serious diseases and stress conditions that compromise physiological homeostasis^11–17^.

Recently, the therapeutic potential of mitochondria transfer and transplantation (MTT) has garnered increasing attention as a novel treatment modality for both acute and chronic diseases^18^. Preclinical studies suggest that exogenous mitochondria can successfully integrate into recipient cells, leading to enhanced ATP production, restoration of redox balance, and improved cellular survival under stress conditions^19^. Promising outcomes have been reported in various conditions, including cardiovascular diseases, neurodegenerative disorders, retinal degenerative diseases, degenerative joint diseases, hearing loss, and respiratory distress syndrome^10,20–25^. MTT is thus emerging as a therapeutic strategy aimed at rescuing dysfunctional cells by augmenting mitochondrial quantity and enhancing overall cellular performance^26^.

The advancement of MTT techniques has largely been driven by breakthroughs in biotechnology—particularly in enhancing mitochondrial uptake efficiency, refining isolation procedures, and developing precise delivery systems^27–29^. The use of nanotechnology-mediated carriers, exosomes, and bioengineered mitochondria has significantly improved the accuracy, biocompatibility, and durability of transplanted mitochondria^30,31^. While biotechnology has played a pivotal role in enabling these developments, ensuring the safety and efficacy of such emerging therapies remains a crucial consideration.

Despite these advancements, several critical challenges still hinder the clinical translation of MTT. Since mitochondria are recognized by the immune system as foreign entities, immune responses may compromise therapeutic efficacy^32^. Moreover, further research into the mechanisms of mitochondrial trafficking is necessary, as recipient cells may exhibit limited capacity for mitochondrial uptake and functional integration^33^. Another key issue is scalability; transitioning from small-scale laboratory experiments to widespread clinical use requires the development of cost-effective, standardized protocols, which are still in progress. Ethical concerns, especially regarding donor mitochondria sourcing, present additional complexities^34^. These limitations underscore the need for a balanced perspective as the field moves forward.

This perspective highlights the transformative potential of MTT in treating mitochondrial disorders by evaluating both the initial successes and the remaining challenges. It emphasizes the importance of integrating mitochondrial biology with other scientific domains—such as bioengineering, cell therapy, and nanotechnology—to overcome current barriers in mitochondrial targeting and to unlock the full therapeutic promise of this approach.

## Current methods for MTT and their limitations

**Fig. 1:**
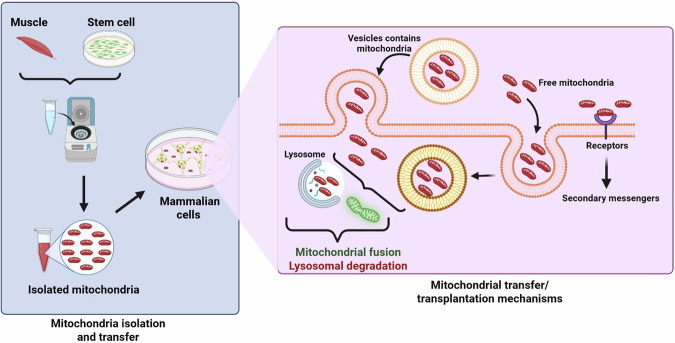
Mitochondria isolation, delivery, and transplantation mechanisms in mammalian cells. The figure depicts the workflow and cell mechanisms involved in mitochondria isolation and transfer into mammalian cells. **Left panel – Isolation and transfer of mitochondria:** Mitochondria can be isolated from in vitro cultured muscle tissues or stem cells through differential centrifugation and purification. The isolated mitochondria obtained are then delivered into in vitro or in vivo mammalian recipient cells. The isolation process must preserve mitochondrial structure and bioenergetic function to support successful transplantation. **Right panel – Mechanisms of mitochondria transfer/transplantation:** Once introduced into the extracellular space, mitochondria can be delivered via various routes. One of the methods involves vesicle-mediated transfer, whereby mitochondria are encapsulated in extracellular vesicles that fuse with the target cell membrane and release their contents. Alternatively, free mitochondria may directly interact with surface receptors or become internalized through endocytosis-like mechanisms. Within the cell, mitochondria have two fates: they can fuse into host mitochondrial networks through mitochondrial fusion, restoring bioenergetic function, or become directed towards lysosomal breakdown if recognized as defective or foreign. Mitochondria ingestion may trigger intracellular transduction cascades by secondary messengers, further impacting recipient cell physiology. Symbols: colors in the figure represent major components: red mitochondria, yellow/orange vesicles, green fused mitochondria, and purple/blue lysosomes. Created in BioRender. Tuncay, E. (2025) https://BioRender.com/uxkej53.

Several mechanisms have been identified through which mitochondria can be transferred between cells via direct transfer approaches, including gap junctions, microvesicles (MVs), and tunneling nanotube (TNTs)^34,35^. TNTs are dynamic, actin-based structures that facilitate long-distance, direct organelle exchange, including the transfer of mitochondria^36,37^. In contrast, gap junctions permit the passage of small molecules—and in certain instances, mitochondria—between adjacent cells^38,39^. Additionally, mitochondria can be encapsulated in MVs and subsequently delivered to target cells^40,41^ (Fig. 1). However, while these naturally occurring strategies are biologically relevant, they present significant limitations for therapeutic applications due to their intrinsic heterogeneity and lack of precision, which may hinder the fine-tuning required for effective MTT.

The maintenance of cellular function is highly dependent on the tight regulation of cellular bioenergetics—a process that can be enhanced through MTT^21^. Specifically, MTT has been shown to improve metabolic regulation and thereby support cellular function^42^. Moreover, it is imperative for the effective transfer of isolated, functional mitochondria to injured cells^43^. The successful transfer of isolated, functional mitochondria to damaged cells is crucial, as it mitigates the adverse effects of mitochondrial dysfunction, which can disrupt critical processes such as protein synthesis, mitochondrial biogenesis, and apoptosis regulation^19^. The bioenergetic impact of MTT is particularly significant, as it provides insight into the mechanisms underlying various disease states and supports the restoration of mitochondrial function and cellular homeostasis^10^.

The process of isolating and transferring mitochondria from donors to recipient cells is referred to as exogenous mitochondria administration or MTT^44–46^. Several delivery routes have been investigated to optimize this process. In systemic injection, mitochondria are introduced into the bloodstream and subsequently absorbed by target tissues^47–49^. While early studies report promising results, this method often suffers from nonspecific distribution and the risk of immune reactions^32^. An alternative approach is direct injection, whereby mitochondria are administered directly into specific organs or tissues—such as the brain or heart^8,50,51^. This method limits systemic dispersion and enables localized therapeutic effects^52–54^. Intranasal delivery, a non-invasive method, has demonstrated the ability to transport mitochondria into the central nervous system, effectively bypassing the blood brain barrier (BBB)^55,56^. While further optimization is needed to improve its efficiency, this strategy holds considerable potential for the treatment of neurodegenerative disorders. Finally, oral mitochondrial administration is currently under investigation^31^. However, this route faces significant challenges, particularly with respect to mitochondrial stability in the gastrointestinal environment and efficient uptake by target cells.

**Fig. 2:**
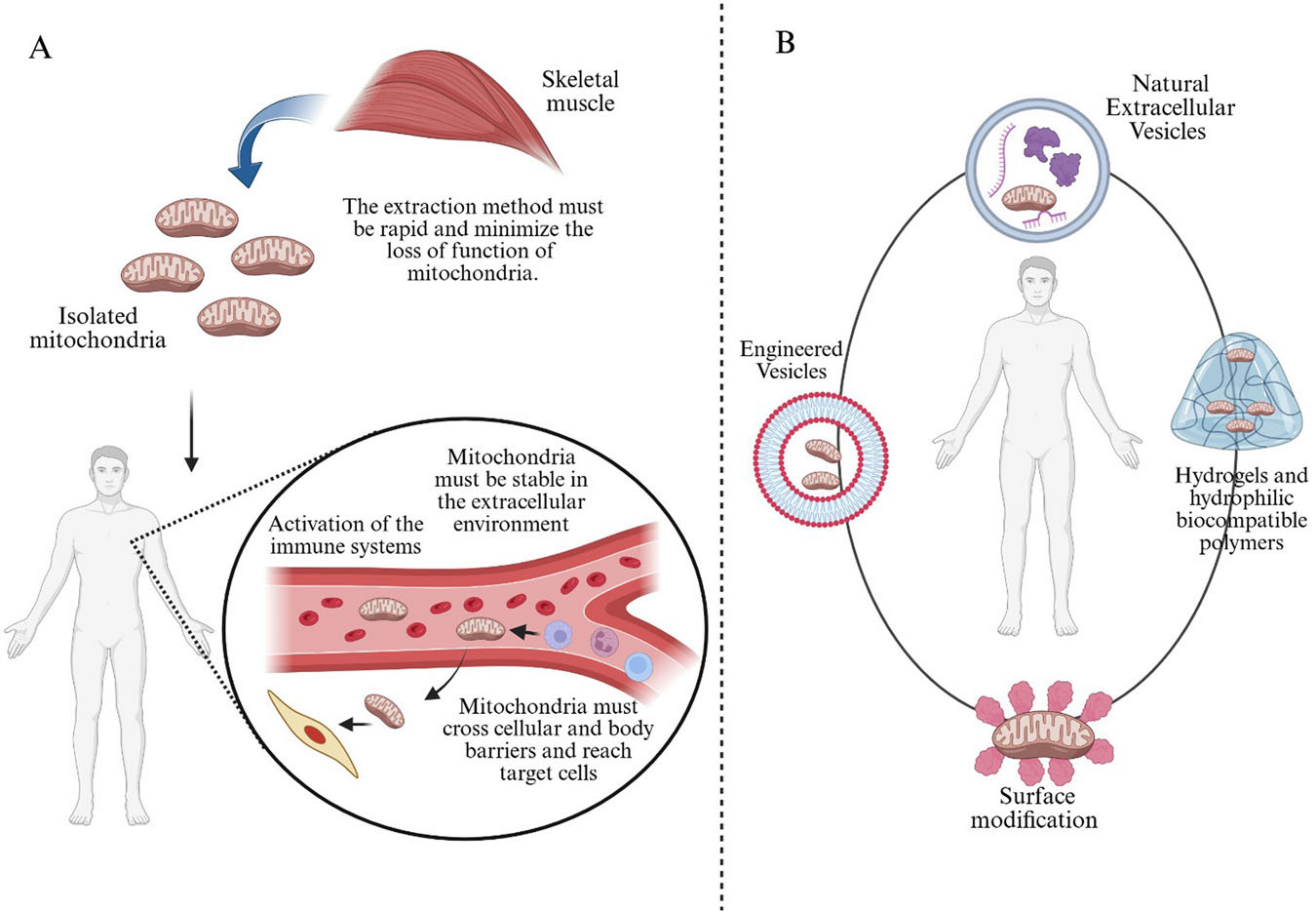
Strategies and challenges in mitochondria isolation and delivery for therapeutic applications. **A** Isolated mitochondria from donor tissues such as skeletal muscle must preserve their functional integrity throughout the course of isolation. Isolation must be achieved rapidly and gently to prevent mitochondrial injury or loss of bioenergetics competence. Following administration into the body, exogenous mitochondria are faced with a series of rigorous challenges. These include risk of immune activation, need for stability in the extracellular environment, and ability to permeate biological barriers—like the vascular endothelium and cell membranes—to access their intracellular targets. **B** To address these challenges and improve delivery efficiency, various strategies have been conceived. These encompass encapsulation in natural extracellular vesicles (e.g., MVs or exosomes), which provide immune protection and target-specific delivery; engineered vesicles, e.g., synthetic nanocarriers or liposomes, which allow for surface functionalization for enhanced stability and targeting; hydrogels and hydrophilic biocompatible polymers, which can be used as scaffolds for local or long-term mitochondrial release; and surface modification techniques, e.g., coating mitochondria with ligands, antibodies, or cell-penetrating peptides, to facilitate cellular uptake and trafficking. Each delivery vehicle is designed to address principal challenges facing mitochondrial therapy, such as immunogenicity, degradation, and poor biodistribution. Color coding and icons are utilized to symbolize crucial components: red mitochondria, red-colored muscle tissue, pink/red vasculature, and purple and blue immune cells. Encapsulation strategies are symbolized by schematic vesicles and hydrogels encapsulating mitochondria, highlighting protective or targeting aspects. Created in BioRender. Nuzzo, D. (2025) https://BioRender.com/b73b997.

Despite the high therapeutic promise of MTT, several critical challenges currently hinder its clinical application^57,58^. As Bertero et al. questioned, MTT may be a “magic bullet” or merely a cause for concern^59^. In fact, MTT has several weak points. Ideally, mitochondria should be isolated quickly (from the patient to the operating room); in fact, a limitation of current MTT methods is the short lifespan of isolated mitochondria^58^, which significantly lose respiratory function after about 2 h. Furthermore, the isolation method must be performed with protocols that minimize the loss of function/vitality and structure of the isolated mitochondria and thus optimize their function^60–62^. The injected mitochondria have to cope with an inhospitable extracellular environment, characterized by a high calcium concentration^59^ or in the case of pathological conditions in which there is exposure to reactive oxygen species (ROS). In addition, injected mitochondria must be stable in the extracellular environment, avoiding aggregation, swelling, and structural changes. It is essential to use viable and functional mitochondria during MTT because, in addition to ensuring mitochondrial functionality in recipient cells, non-viable or damaged mitochondria can release damage-associated molecular patterns that lead to activation of the immune system^44^. Injected mitochondria must be able to cross cellular and body barriers and reach target cells^60,61^. In fact, it has been shown that only a small proportion (10%) of the injected mitochondria reach the cells^53^, and the transfer is not specific for target cells (Fig. 2A). In this context, the surface charge of isolated mitochondria could reduce the interaction with target cell membranes. Finally, once inside the cells, the mitochondria must be able to reach the cytoplasm, avoiding the lysosomal degradation pathway, and integrate into the mitochondrial network of the recipient cell.

It remains unclear what processes are involved in fusing functional mitochondria with the recipient mitochondrial network or, given the highly dynamic nature of mitochondria, whether and how the mitochondrial cargo degrades/diffuses into the cytoplasm. It is also important to determine the survival or functional period of transplanted mitochondria or their components, especially in a cellular environment often characterized by mitochondrial dysfunction.

## Biotechnology in mitochondria transfer and transplantation

Biotechnology has been shown to be an effective tool for overcoming the inherent limitations of the MTT. However, reaching functional improvements or answers to all the above requires a combination with fresh advances from within the biotechnology realm. The few promising emerging technologies, which will certainly contribute to enhancing mitochondrial targeting and delivery, hence addressing these challenges, are biotechnology and engineered delivery systems^63^ (Table 1). These advances aim to enhance the specificity, efficiency, and safety of mitochondria delivery while minimizing toxicity and immune activation. In doing so, they pave the way for the development of scalable and commercially viable therapeutic applications. In the recent years, several biotechnological strategies have been proposed to enhance mitochondrial targeting and protection. These include surface modification of isolated mitochondria, the use of extracellular vesicles (EVs) or artificial vesicles encapsulating mitochondria, the development of hydrogels designed to retain and release functional mitochondria and functionalization of mitochondrial surfaces with hydrophilic, biocompatible polymers (Fig. 2B). These approaches provide a protective microenvironment for mitochondria during transit, shielding them from enzymatic degradation, immune detection, and oxidative damage. Furthermore, these delivery vectors can be engineered with targeting ligands, enabling enhanced interaction with specific cell types and allowing for controlled release of mitochondrial cargo. Collectively, these innovations represent a significant advancement toward harnessing the full therapeutic potential of MTT.

**Table 1:** Summary table of mitochondria delivery strategies in different pathologies and model cell lines, their therapeutic effect and the efficiency of MTT in comparison to free mitochondria

Mitochondria delivery strategies	Pathologies and cellular models	Mitochondria transfer efficiency	Biological effects	Reference
Mitochondria conjugated with Pep-1	Parkinson’s disease in vitro and in vivo models	60.5% of the cells received allogeneic mitochondria transferred by Pep-1, compared to 14.5% with allogeneic naked mitochondria	Anti-apoptotic effect, prevention of oxidative stress, and improvement in locomotive activity	64
Mitochondria functionalized with dextran and TPP	Breast and heart cancer cells	Approximately threefold increase in mitochondrial internalization compared to uncoated mitochondria	Reduction of reactive oxygen species	69
PEP peptide and TPP complex to mitochondria	Myocardial ischemia–reperfusion injury	Intravenous injection of mitochondria showed no therapeutic effect compared to PEP-TPP mitochondria	Reduction in cell apoptosis, inflammatory response and infarct size	70
TAT-dextran-coated mitochondria	Reperfusion injury models in cultured cardiomyocytes	182.8% increase in mitochondrial TAT-dextran transfer over free mitochondria	Anti-apoptotic effect and prevention of oxidative phosphorylation impairment after oxidative stress	65
Oral administration of nanomotorized mitochondria	Mouse models of acute and chronic ischemic heart disease	The nanomotorized system enabled 7.9% of the mitochondria-containing system to reach cardiac tissue, compared to 1.0% for non-motorized mitochondria	Prevents progression of acute and chronic ischemic heart disease	31
Microvesicles	In vitro model of ischemic myocardium (hypoxia-damaged cardiomyocytes)	Isolated mitochondria showed no effect compared to mitochondria delivered via microvesicles	Restoration of bioenergetics	73
Enclosed mitochondria with DOTAP/DOPE (AM-Mito)	Cerebral ischemia-reperfusion	In AM-Mito treated cells, mitochondrial fluorescence increased twofold compared to treatment with bare mitochondria	Neuroprotection and reduction in cerebral infarct size	86
Pluronic F127 hydrogel-coated mitochondria (PF127-Mito)	Myocardial ischemia-reperfusion injury	PF127-Mito group showed significantly greater internalization of mitochondria compared to Naked-Mito	Improves mechanical and metabolic capabilities of cardiomyocytes	90

## Surface modification of mitochondria

Surface modification with cell-penetrating peptides (CPPs) enhances the precision of mitochondria delivery and internalization, which is critical for increasing the efficiency of MTT. CPPs are typically short, positively charged peptides that facilitate interactions with negatively charged cell membranes, enabling their entry into cells and subsequent delivery of cargo. They have been widely used to enhance the cellular uptake of various agents, including nanoparticles, DNA, and proteins. A well-known CPP, the HIV-1 TAT protein, has been shown to mediate the import of functionally active mitochondrial enzymes—such as complex I subunits—via covalent coupling, thereby promoting the restoration of essential cellular functions. Another CPP, Pep-1, can translocate cargo through non-covalent self-assembly. Once inside the cell, cargo is released via an endocytosis-independent mechanism. Both TAT and Pep-1 have demonstrated efficacy in facilitating mitochondrial uptake and release within cells^64,65^.

Chang et al investigated the effect of mitochondrial conjugation with the peptide transporter Pep-1, Pep-1 mediated mitochondria delivery (PMD), on mitochondria transfer efficiency, compared to cell free mitochondria. The Pep-1/mitochondria complex was prepared at a weight ratio of 1750:1 by incubation at 37 °C for 30 min^66^. This modified system was tested in various disease model, including neurotoxin (6-hydroxydopamine, 6-OHDA)-induced PC12 cells, Parkinson’s disease (PD) rat models, a cybrid cell model of mitochondrial myopathy, encephalopathy, lactic acidosis, and stroke-like episodes^64,67,68^.

To evaluate whether Pep-1 labeling alters mitochondrial structure, mitochondria exposed to varying concentrations of Pep-1 were examined via transmission electron microscopy (TEM)^67^. No changes in overall mitochondrial morphology were detected, indicating that Pep-1 preserves mitochondrial integrity. However, an increase in outer membrane contact—indicative of membrane fusion—was observed. Membrane fusion was reported at 34%, 62%, and 77% following treatment with 5, 25, and 50 μM Pep-1, respectively, compared to 11% observed in the controls^67^. The authors attributed this to Pep-1’s high affinity for lipid membranes, which allows for membrane integration and enhanced mitochondrial fusion. Notably, Pep-1 promoted the formation of tubular mitochondrial networks without affecting mitochondrial-shaping proteins and enhanced mitochondrial function both in vitro and in vivo^67^. Although increased membrane fusion may support integration into the host mitochondrial network, it may also contribute to mitochondrial clustering. Therefore, reducing this fusion effect could potentially improve the efficiency of Pep-1–mediated MTT. Supporting this, the same group demonstrated significantly higher transfer efficiency using Pep-1–conjugated mitochondria compared to unmodified mitochondria. In PC12 cells, 60.5% (PMD) and 56.8% (xPMD) of cells showed exogenous mitochondrial signals, versus 14.5% and 18.02% in the naked allogenic and xenogenic mitochondria groups, respectively^64^. In PD mouse models, mitochondria were administered directly into the medial forebrain bundle^67^ or intranasally^68^. While Pep-1–mediated MTT showed greater efficacy overall, the difference was statistically insignificant via the intranasal route. Nonetheless, intranasal P-Mito administration significantly suppressed inflammatory cytokine levels^68^.

Another approach to improving MTT involves mitochondria functionalized with dextran (Dex) and triphenylphosphonium (TPP). TPP possesses a substantial lipophilic component that facilitates its interaction with the phospholipid bilayer, thereby promoting efficient cellular uptake. This process, driven by hydrophobic interactions, occurs independently of cell type. Therefore, TPP associated with mitochondrial membranes may come into direct contact with cell membranes, resulting in increased cellular accumulation. Dex is a polysaccharide, used in several biomedical applications, that has been shown to improve the uptake of nanomaterials in vivo^28,69^. Isolated mitochondria functionalized with Dex-TPP have been shown to protect the mitochondria and facilitate cellular internalization^28,69^.

The optimal Dex-TPP mitochondria ratio was determined to be 1.9:1 based on mitochondrial respiration analysis^69^. Using Oroboros high-resolution respirometry, the authors evaluated the respiratory performance of both uncoated and coated mitochondria. The Dex coating placed the isolated mitochondria into a state of metabolic dormancy, effectively safeguarding their respiratory function. This approach showed a reduction in ROS generation^28^ and facilitated cellular internalization compared to uncoated mitochondria in cardiac cells^69^. Indeed, the results demonstrated efficient internalization of coated mitochondria in breast cancer and cardiac cell lines, showing approximately a threefold increase compared to uncoated mitochondria^69^. Furthermore, since mitochondria are known to be highly negatively charged, the Dex-TPP coating could have a positive effect on internalization by reducing the negative charge^69^. In fact, positively charged biomaterials are known to have higher internalization rates due to electrostatic interactions with the negatively charged cell membrane^69^. MTT has emerged as a promising approach to mitigate myocardial ischemia-reperfusion (IR) injury. However, its clinical application remains constrained by the need for direct mitochondria injection into the myocardium, typically via invasive catheter- or sternotomy-based methods. As an alternative, targeting peptides offer a molecular strategy to deliver mitochondria to the damaged myocardium through systemic intravenous administration. Recently, Sun et al. investigated the efficacy of intravenous MTT in myocardial IR injury, employing techniques such as echocardiography, infarct size measurement, and evaluation of pro-apoptotic markers, including cleaved caspase-3 and the BAX/BCL-2 ratio. Their findings revealed no therapeutic benefit from intravenous MTT; instead, it amplified the expression of pro-inflammatory factors. Consequently, the authors concluded that intravenous mitochondria delivery lacks efficacy in alleviating myocardial IR injury^70^. To address this limitation, the authors devised an innovative approach to transfer healthy mitochondria into the injured myocardium^70^. They utilized CSTSMLKAC (PEP), a polypeptide (Cys-Ser-Thr-Ser-Met-Leu-Lys-Ala-Cys) previously shown to exhibit high selectivity for ischemic myocardium. By conjugating the PEP peptide and a PEP-TPP complex to mitochondria, they demonstrated that the transplanted mitochondria efficiently dissociate from the PEP-TPP-mitochondrial conjugate and are internalized by cardiomyocytes, facilitated by endothelial cells. This targeted delivery significantly reduced cell apoptosis, attenuated the inflammatory response, and decreased infarct size, highlighting a potential advancement in MTT for myocardial IR injury^70^.

Dex has also been conjugated to the TAT peptide, which is characterized by an arginine-rich sequence and a strong positive charge. The conventional reductive amination method was performed to conjugate TAT peptides to Dex, termed TAT-dextran. TAT-dextran was used to coat the isolated mitochondria. To determine whether TAT-dextran adversely affected mitochondrial respiratory function, respirometry was used; parameters were nearly the same between isolated mitochondria and TAT-dextran-coated mitochondria (TAT-Dex-Mito), indicating that such functionalization has no adverse effects on cellular respiration. TAT-Dex-Mito showed a significantly higher level of cellular uptake, of exogenous mitochondria in primary cultures of neonatal rat cardiomyocytes (NRCM) by flow cytometric analysis (about 182.8%, versus Mito group). MTT showed an anti-apoptotic effect and prevented the impairment of oxidative phosphorylation in mitochondria after oxidative stress in NRCMs, compared to simple mitochondria transfer^65^.

A novel approach has recently been proposed, involving the oral administration of nanomotorized mitochondria to enhance the efficiency of therapeutic delivery^31^. Isolated mitochondria were coated with L-arginine-derived methacrylate (M-Arg), a substrate of inducible nitric oxide (iNOS), and the ROS-responsive cross-linking agent diselenide by polymerization reaction to form the nanomotor component (NM) of the mitochondria (NM/Mito). In addition, the CM component, represented by cardiomyocyte membrane fragments, was asymmetrically added to the surface of the NM/Mito by electrostatic interaction, resulting in CM/NM/Mito. Finally, the latter was encapsulated to obtain CM/NM/Mito@Cap for oral administration to mouse models of acute and chronic ischemia heart disease (IHD). CM/NM/Mito@Cap was designed to remain intact in the presence of gastric acid and dissolve in the intestine^31^. Characterization of the physicochemical properties in conjunction with bioactivity analyses demonstrated the efficacy of the mitochondrial modification, thereby confirming the preservation of the original biological functions of the mitochondria. The CM component can promote the rapid endocytosis of mitochondria by intestinal epithelial cells, followed by exocytosis into the bloodstream. Subsequently, CM/NM/Mito delivered to the blood circulation are transported by the chemotaxis of the NM component to the damaged site of the heart, where they are strongly retained in the damaged cardiomyocytes^31^. The high levels of ROS and iNOS at the lesion site served as chemoattractants for CM/NM/Mito the ability to selectively target the ischemia heart following oral administration was evaluated in a mouse model of acute and chronic IHD. Rats with acute IHD were administered Mito@Cap, NM/Mito@Cap or CM/NM/Mito@Cap, which contained 1 × 10^9^ MitoTracker-labelled particles^31^. The results demonstrated that oral administration of CM/NM/Mito@Cap enabled approximately 7.9% (oral dose rate per gram of cardiac tissue) of the system to reach cardiac tissue six hours after administration, in comparison to 1.0% Mito@Cap and 1.1% NM/Mito@Cap. Furthermore, CM/Mito (lacking a NM component, i.e., without chemotactic capacity) exhibited suboptimal cardiac penetration and retention, with a cardiac delivery efficiency of 1.7% of the oral dose per gram of cardiac tissue^31^. Nanomotorised mitochondria accumulated in damaged heart tissue regulate cardiac metabolism and prevent progression of IHD^31^.

## Natural extracellular vesicles

EVs are a heterogeneous population of secreted lipid bilayer-enclosed vesicles containing nucleic acids, proteins, metabolites, metabolic enzymes, lipids, and even entire organelles such as mitochondria^71^. Mitochondria transfer is a naturally occurring physiological process. For example, in response to neuronal damage induced by ischemic stroke, astrocytes can transfer their mitochondria to neurons through mitochondria-containing vesicles, thereby contributing to neuroprotection. Certain subtypes of EVs, such as MVs, can encapsulate all mitochondrial components, thereby preserving their functional integrity. Indeed, several studies have confirmed the presence of intact, functional mitochondria within EVs^71^. It has been demonstrated that the lipid membrane of EVs plays a critical role in protecting their cargo, maintaining mitochondrial integrity and function over time^72^. The extracellular environment of injured tissue can compromise the viability of exogenously administered mitochondria. In one study, the viability of free mitochondria was compared to that of mitochondria encapsulated in EVs under varying concentrations of Ca²⁺ and ROS. The results showed that EV-encapsulated mitochondria maintained their functionality for up to 24 h in the presence of 2 mM Ca²⁺ and 100 mM H₂O₂, while free mitochondria did not, suggesting enhanced resistance to calcium overload and oxidative stress when mitochondria are encapsulated within EVs^73^. Thus, mitochondrial encapsulation in EVs offers a strategy for prolonged preservation of mitochondrial function, thereby improving MTT efficiency. In an in vitro model of ischemic myocardium (hypoxia-injured cardiomyocytes), bioenergetic parameters were significantly restored as early as three hours after treatment with MVs (1.0 × 10⁸/mL). In contrast, isolated mitochondria containing 100-fold higher levels of mitochondrial proteins (VDAC and COX IV) than MVs failed to exert any detectable effect even after 24 h^73^.

MVs can deliver intact mitochondria to brain endothelial cells (BECs), resulting in a 200-fold increase in ATP levels compared to untreated cells^74^. Furthermore, EV-mediated MTT promotes mitochondrial biogenesis and ATP production, thereby mitigating doxorubicin-induced cardiotoxicity in cardiomyocytes^75^. Liu et al. utilized vesicles to transfer healthy mitochondria from astrocytes to neurons, effectively alleviating mitochondria-mediated neuronal damage^29^. Specifically, engineered macrophage-derived exosomes loaded with a heptapeptide, a Drp1/Fis1 interaction inhibitor, not only reduced mitochondrial damage in astrocytes but also facilitated the release of healthy astrocytic mitochondria via EVs and their subsequent transfer to neurons, thereby protecting against neuronal mitochondrial dysfunction^29^.

Additionally, EVs combined with exogenous heat shock protein 27 (HSP27) represent a promising strategy for synergistically enhancing BEC survival and preserving tight junction integrity in an in vitro BBB model, aimed at preventing long-term neurological dysfunction following ischemic stroke. The EVs/HSP27 mixture was prepared by incubating EVs derived from human cerebral endothelial cells with 2 μg of HSP27 at a protein ratio of 10:1 (EV:HSP27) at room temperature. This formulation did not significantly alter the particle size compared to naïve EVs (~ 225 nm) and remained cytocompatible for up to 72 h on primary BECs. Despite the negative surface charge of both EVs and native HSP27 at physiological pH (7.4), the EV/HSP27 complexes exhibited approximately 20% binding efficiency. The authors noted that the weak, non-invasive interaction between HSP27 and EVs serves to preserve the lipid membrane integrity and maintain the functionality of EV-contained mitochondria—an outcome that would likely be compromised by conventional HSP27-loading methods such as freeze-thaw cycles or sonication^76^.

To further exploit the potential of MVs in MTT under post-ischemic conditions, a study demonstrated that encapsulation of mitochondria within vesicles can be enhanced by stimulating mitochondrial biogenesis in donor cells. Specifically, the use of resveratrol to activate PGC-1α, a master regulator of mitochondrial biogenesis, resulted in increased MV secretion from BECs and a corresponding elevation in ATP levels in recipient BECs compared to naïve MVs from non-activated cells^77^.

Recently, mitochondria delivered by EVs derived from neural stem cells were shown to restore mitochondrial function in a mouse model of experimental autoimmune encephalomyelitis (EAE), an established model of multiple sclerosis^78^. The authors highlighted that the presence of mitochondria within EVs was essential for the observed therapeutic effect in vivo. Notably, the administration of mitochondria-depleted EVs did not lead to significant clinical improvements in EAE mice. Furthermore, mechanistic studies revealed that EV uptake by mononuclear phagocytes primarily occurred via endocytosis, as evidenced using pharmacological inhibitors targeting phagocytosis and endocytosis pathways. Once internalized, EVs were shown to integrate into the host mitochondrial network^78^.

These findings raise an important question: Is mitochondrial integrity and/or functionality a prerequisite for the protective effects of EVs? Some studies have shown that the transfer of mitochondria damaged by oxidative stress from astrocytes to neurons may not be beneficial; on the contrary, it may exacerbate neuronal damage and worsen cerebral ischemic injury^71,79^. Although the presence of mitochondrial components—such as depolarized or structurally incomplete mitochondria—within EVs has been documented, this does not inherently indicate a loss of biological activity^80^. For instance, Crewe et al. found that adipocytes exposed to oxidative stress release EVs enriched in damaged mitochondrial components^81^. These EVs were internalized by neighboring cells, triggering enhanced antioxidant responses. Additionally, the release of damaged mitochondria was suggested to serve as a protective mechanism to reduce mitochondrial burden in stressed maternal adipocytes^82^.

Beyond intact or damaged mitochondria, EVs may selectively carry mitochondrial biomolecules, including mitochondrial DNA (mtDNA), proteins, and mitochondrial RNA (mtRNA)^71^. These mitochondrial components can exert various effects, potentially contributing to disease progression or acting as diagnostic biomarkers. Due to this heterogeneity, the functional implications of mitochondrial content within EVs remain an active area of investigation.

Moreover, the molecular mechanisms underlying the release of mitochondria or their components from EVs upon uptake by recipient cells are still poorly understood. EV internalization may occur via endocytosis or direct fusion with the plasma membrane^83^. The membrane composition of EVs—including phospholipids, integrins, and tetraspanins—may facilitate these uptake processes by promoting membrane fusion or engaging pinocytic pathways^84^.

Interestingly, in a mouse model of transient cerebral ischemia, astrocyte-derived mitochondria were shown to enter adjacent neurons via EV-mediated transport, contributing to neuronal survival. The knockdown of CD38 using short interfering RNA reduced the release of extracellular mitochondria and worsened neurological outcomes, emphasizing the functional relevance of this pathway^85^. Notably, EVs can cross the BBB, with exosomes expressing CD46 and intercellular adhesion molecule-1—both known to interact with BECs. These findings suggest that EVs transporting functional mitochondria may utilize similar mechanisms to traverse the BBB and exert neuroprotective effects^85^. A deeper understanding of the intracellular trafficking of EV-carried mitochondria and the fate of EVs themselves is essential to further elucidate the mechanisms of mitochondria transfer. This knowledge could pave the way for enhancing the therapeutic efficiency of EV-mediated mitochondria delivery.

## Engineered Vesicles

Engineered vesicles play a crucial role in MTT by serving as vectors for the targeted delivery of mitochondria. Encapsulating mitochondria within liposomes enhances the efficiency and specificity of mitochondria transfer by protecting the organelles during delivery and ensuring their successful internalization by target cells. Importantly, this encapsulation prevents the exposure of mitochondrial components that may otherwise provoke undesirable cellular responses or immune activation^86^. Functionalization of liposomes with specific targeting ligands—such as antibodies or peptides—further facilitates the selective delivery of mitochondria to cell types or tissues. Coating the mitochondrial surface with a lipid membrane significantly improves delivery efficiency and cellular uptake. For example, mitochondria encapsulated within a DOTAP/DOPE lipid bilayer (referred to as artificial membrane-coated mitochondria, or AM-Mito) demonstrated an encapsulation efficiency of approximately 86%^86^. The resulting AM-Mito particles exhibited an average size of ~846.4 nm. Importantly, key mitochondrial proteins—including TOM40, ATP5a, ACADM, HSP60, and COX IV—as well as mitochondrial membrane potential, were preserved following encapsulation. TEM confirmed that mitochondrial morphology remained intact post-coating, and ATP content was comparable between naked mitochondria and AM-Mito. Furthermore, flow cytometry analysis using JC-1 dye indicated that mitochondrial membrane potential was slightly higher in AM-Mito than in uncoated mitochondria, suggesting improved bioenergetics stability^86^. The lipid coating also reduced the negative surface charge of the mitochondria, thereby promoting interaction with cellular membranes and enhancing uptake. In primary mouse neurons, fluorescent labeling with MitoTracker Deep Red revealed a twofold increase in intracellular fluorescence intensity in cells treated with AM-Mito compared to those treated with naked mitochondria after just three hours of incubation. Additionally, AM-Mito conferred significant neuroprotection against oxygen-glucose deprivation in vitro^86^. In vivo studies further validated the therapeutic potential of AM-Mito. Intravenous administration of AM-Mito immediately following focal cerebral IR injury led to increased mitochondrial presence within brain tissue and a marked reduction in infarct volume, confirming the neuroprotective efficacy of this engineered delivery system^86^.

Synaptosomes—synaptic vesicles derived from rat brain terminals (isolated via sucrose density gradient ultracentrifugation) and containing mitochondria—have been employed as natural vesicle-based carriers for MTT. Although the authors did not directly compare the efficacy of free versus synaptosome-encapsulated mitochondria, they demonstrated that synaptosomes exhibit neuron-specific uptake, mediated by surface proteins that act as targeting agents for neuronal cells. These vesicles successfully restored mitochondrial viability and cellular activity in damaged neurons^87^ Notably, the authors also showed that mitochondria encapsulated within synaptosomes remained viable after freezing, suggesting that these structures could serve as a stable, on-demand mitochondrial reservoir for transplantation^87^. A membrane fusion-based mitochondria delivery strategy was also developed using fusogenic mitochondrial capsules (FMCs). These were constructed from a combination of lipids including a neutral lipid (phosphatidylethanolamine, PE), a cationic lipid (DOTAP), and an aromatic lipid (Liss Rhod PE), forming three types of liposomes—FMC0, FMC1, and FMC2—differentiated by varying DOTAP concentrations. The structural stability of FMCs was assessed over time in comparison to unencapsulated mitochondria. Results indicated that the size of uncoated mitochondria significantly increased from day 3 onward, suggesting degradation or dysfunction, while FMCs maintained a stable size over time^88^. As particle stability alone is insufficient to confirm mitochondrial viability, the expression levels of mitochondrial cytochrome c (CytoC) were assessed. CytoC release into the cytosol is a hallmark of mitochondrial dysfunction and apoptosis. FMC2 demonstrated a twofold increase in CytoC expression compared to naked mitochondria, indicating enhanced structural stability and preservation of mitochondrial function. Furthermore, FMCs were shown to be cytocompatible and to reduce mitochondrial degradation by evading lysosomal digestion and mitophagy, likely due to a membrane fusion-based internalization pathway^88^. Compared to uncoated mitochondria, FMCs exhibited faster and more efficient uptake into chondrocytes. In vitro and in vivo experiments further revealed that FMC treatment downregulated the expression of inflammatory cytokines and matrix metalloproteinase-13, while upregulating extracellular matrix components and promoting cartilage regeneration^88^.

By conjugating targeting ligands—such as peptides, antibodies, or other biomolecules—to the liposomal surface, researchers can selectively guide mitochondria delivery vehicles to specific receptors on diseased cells, enhancing both targeting precision and therapeutic efficacy. Additionally, the lipid bilayer structure of liposomes mimics the natural mitochondrial membrane, improving compatibility and fusion with recipient cells. Advanced formulations, such as PEGylated liposomes, offer further advantages by extending systemic circulation time through reduced immune clearance, thereby increasing the likelihood of successful mitochondria delivery to target tissues.

More recently, mitochondria-containing and lung-targeting liposomes loaded with a ROS scavenger—referred to as LMR (lung-targeted mitochondria with ROS scavenger)—were developed. These LMRs were prepared by extracting mitochondria from AC16 cells and co-fusing them with liposomes loaded with the ROS scavenger Tempol. The lipid film was composed of DDAB, cholesterol, and DSPE-PEG2000-Tempol, and mitochondria were suspended to hydrate the lipid film using a solvent evaporation technique. This system was designed to mitigate oxidative stress associated with acute respiratory distress syndrome^89^. The LMRs exhibited preferential localization in lung tissue compared to free mitochondria. The authors proposed that this tropism may result from electrostatic interactions between negatively charged erythrocytes and the positively charged LMRs, leading to transient aggregation and pulmonary retention via capillary perfusion^89^. While liposomal surface modifications and co-loading strategies using mito-protective molecules remain underexplored, these approaches hold promises for enhancing both delivery efficacy and mitochondrial survival in MTT applications.

## Hydrogels and hydrophilic biocompatible polymers

Mitochondria-loaded hydrogels and mitochondria coated with biocompatible hydrophilic biopolymers have been explored to protect mitochondria from external environmental stress and to enhance their local diffusion and uptake by recipient cells. Several types of polymers have been investigated for their role in mitochondria delivery. Pluronic F127 (PF127) hydrogel, for example, has been used to encapsulate mitochondria (PF127-Mito) for the treatment of myocardial IR injury^90^. The physical state of PF127 is temperature-sensitive: it remains in a liquid phase at low temperatures but transitions to a gel form at body temperature, facilitating the resuspension of mitochondria in PF127 prior to injection into myocardial tissue. Notably, no toxic effects were observed when adult primary cardiomyocytes or H9C2 cells were incubated with 15% PF127, and no myocardial apoptosis was detected in mice after injection of PF127 using the TUNEL assay. The hydrogel’s pore size ranged from 10 to 20 μm, while encapsulated mitochondria measured between 0.4 and 1.5 μm^90^. The PF127 hydrogel significantly enhanced mitochondrial membrane integrity under high calcium conditions, simulating a transplantation environment. Compared to naked mitochondria, PF127-coated mitochondria were better protected from several transplant-related damages, such as swelling or disruption of mitochondrial integrity. Both PF127-Mito and naked mitochondria reached peak internalization in cardiomyocytes within 1–3 h. However, the PF127-Mito group exhibited a substantially greater mass of internalized mitochondria compared to naked mitochondria. In vitro studies demonstrated that PF127-supported MTT significantly improved the mechanical and metabolic functions of cardiomyocytes following hypoxia-reoxygenation injury. In vivo, PF127-Mito improved cardiac function, reduced infarct size, and ameliorated adverse myocardial remodeling in mice following IR injury, offering a promising strategy to protect mitochondria during transplantation and promote cardiac repair post-IR^90^. Patel et al. used hyaluronic acid and methylcellulose to prepare an erodible thermogelling hydrogel for local mitochondria delivery to injured spinal cord tissue^27^. The results showed no significant differences in mitochondrial membrane potential when mitochondria were encapsulated in the hydrogel, indicating that the hydrogel did not adversely affect mitochondrial integrity. Seventy percent of the encapsulated mitochondria were released within 60 min at 37 °C, and the mitochondria exhibited greater respiratory capacity compared to their unloaded counterparts, suggesting that the hydrogel helped preserve mitochondrial function during delivery^27^.

Electroconductive hydrogels based on alginate have also been employed to encapsulate isolated mitochondria. These hydrogels serve as excellent platforms for cell and organelle transplantation, aiding the regeneration of ischemic cardiac tissue^91^. Results showed that hydrogels preserved the integrity and morphology of the encapsulated mitochondria. Injection of mitochondria-loaded hydrogels into myocardial tissue resulted in significant restoration of left ventricular thickness compared to the infarct zone, 14 days after injection. Although the authors did not compare the uptake of mitochondria encapsulated in the hydrogel with free mitochondria in cardiomyocytes, they observed that the hydrogel prolonged the local density of transplanted mitochondria at the site of injury, thereby facilitating targeted delivery and sustained release at the infarct site^91^.

Additionally, degalactosylated xyloglucan hydrogels (DxH) have been developed as carriers for mitochondria delivery^92^. Xyloglucan consists of a β-(1,4)-D-glucan backbone, which is partially substituted with α-(1,6)-D-xylose residues, some of which are further substituted with β-(1,2)-D-galactoxylose. A thermally responsive version of xyloglucan, known as partially degalactosylated xyloglucan (dXG), can be generated through enzymatic treatment with fungal β-galactosidase, which removes over 35% of the galactose residues. When the galactose removal ratio is between 35% and 50%, dXG can form hydrogels from aqueous solutions at concentrations exceeding 1% w/v at body temperature. To assess the effect of DxH on mitochondrial integrity, the mitochondrial membrane potential of mitochondria encapsulated in the hydrogel was monitored. Notably, the presence of DxH improved the mitochondrial membrane potential compared to free mitochondria, indicating the preservation of mitochondrial health within the hydrogel. This effect may be attributed to xyloglucan’s ability to enhance energy metabolism. Furthermore, superoxide species generated due to oxidative changes in the mitochondria were measured. Mitochondria encapsulated in the hydrogel showed fluorescence intensity like that of free mitochondria, suggesting that the hydrogel did not induce oxidative stress in the mitochondria. The hydrogel also provided several protective benefits, shielding mitochondria from external environmental stress and oxidative damage. Although the authors did not directly compare the transfer efficiency of free mitochondria with that of mitochondria encapsulated in the hydrogel, the gel facilitated the sustained release of viable mitochondria and enabled localized delivery to the target cells^92^.

## Expert opinion on biotechnological applications in MTT

In view of the limitations of naked MTT, there is a strong tendency for scientific research to move towards the use of biotechnology. The data presented herein indicate that the systems utilized do not modify the structure or functionality of mitochondria. However, this aspect is not always exhaustively addressed. Indeed, the effects that these systems have on mitochondrial activity are analyzed with reduced techniques and should be studied in more detail. This is particularly crucial in approaches involving superficial modification of mitochondria by binding with molecules or peptides. Furthermore, a significant proportion of extant studies have focused on the efficacy of MTT in the recovery of mitochondrial dysfunction, utilizing both in vivo and in vitro models of pathologies. However, there is a paucity of detailed studies on the safety of the treatment. Even where such studies exist, they are not well investigated or detailed. It is imperative to consider the general effect on different organs and tissues of the administration of these new formulations in in vivo models. Moreover, a significant proportion of studies to date have concentrated exclusively on the monitoring of the short-term effects. Nevertheless, to ascertain the true effectiveness and curative potential of such biotechnological therapies, it is essential to study the effects of MTT over a more protracted period. Such research is also required to determine whether regular infusion treatments are necessary for successful management of the condition.

EVs have been found to contain intact mitochondria or mitochondrial fragments in addition to various molecules, including mtDNA, mtRNA, proteins, metabolites, metabolic enzymes, and lipids. Consequently, the therapeutic effect of EVs may be attributable to non-mitochondrial components. To attribute the therapeutic effect to the transferred mitochondrial components, experiments should be carried out in which this can be distinguished from other components. For instance, the inactivation of mitochondria within EVs offers a compelling approach, as it establishes a correlation between the therapeutic effect of EVs and functional mitochondria^76,78^. However, given the heterogeneity of EVs (which can contain whole mitochondria, fragments of mitochondria, or molecules derived from mitochondria), the identification and isolation of the different EVs according to their content to standardize and control their effect on the efficiency of MTT will be laborious and will require further investigation.

From this standpoint, the employment of engineered vesicles facilitates a superior approach, as the content can be meticulously regulated, and the manufacturing process is more amenable to industrial-scale production. Furthermore, the rigorous regulatory testing to which numerous drug delivery systems have already been subjected for clinical approval suggests that their utilization in MTT could accelerate the application of MTT in the clinic.

An important and under-researched factor is to understand whether mitochondria from different tissues or cells have different structural and functional properties (in terms of cellular respiration and enzymatic activity) that can improve the therapeutic efficacy of MTT. In this sense, biotechnology could help to create engineered mitochondria (artificial mitochondria) that could be more efficient and more resistant to adverse events that occur during transplantation.

In the domain of tissue engineering, the utilization of mitochondria-loaded hydrogels has emerged as a prominent research focus. These hydrogels, engineered to house mitochondria, are injected near injured tissue^27^. The studies undertaken thus far have demonstrated the capacity of these hydrogels to safeguard the integrity of mitochondrial components within the extracellular environment. However, their mechanism of action involves the localized release of mitochondria, rather than the targeting in specific cells, as observed in other strategies.

Among the various approaches mentioned, the co-delivery of mitochondria with mitochondrial protective molecules^85,88^, such as antioxidant molecules, is worthy of particular attention. This approach aims not only to replace dysfunctional mitochondria, but also to create an intracellular environment that supports the optimal performance of the transferred organelles. However, the literature on this subject is limited, and while the strategy is promising and innovative, further research is required to expand upon it. Finally, the cost of manufacturing and implementing mitochondria delivery systems may limit their accessibility to a wider population. Consequently, a cost analysis should be conducted to assess the affordability of the treatment, a crucial aspect that is currently lacking in the extant literature.

## Challenges facing nanotechnology-based MTT

### Biocompatibility and immune response

Nanoparticles are widely used in medicine and are emerging as a promising class of therapeutics. However, their biocompatibility and potential to provoke immune responses remain critical concerns^93^. One of the foremost challenges in nanotechnology-based MTT is ensuring compatibility with host tissues while effectively managing the immune response^94^. Notably, nanoparticles have been shown to impair mitochondrial function in targeted cells and influence key mitochondrial signaling pathways, including those involved in apoptosis and autophagy^95,96^. Such effects can potentially increase the host’s susceptibility to disease^94^. While the therapeutic delivery of exogenous mitochondria hold significant promise, introducing foreign or even autologous mitochondria into host tissues raises important immunological concerns. Interestingly, some studies have reported that MTT does not elicit a notable immune response in in vivo models^97,98^. Conversely, other findings indicate that even a single injection of isolated mitochondria can induce an immune response characterized by the activation of pro-inflammatory cytokines and chemokines in endothelial cells, suggesting that mitochondria may act as a potent immunostimulator^32^. To address these challenges, several strategies have been proposed, such as PEGylation, which can reduce the immunogenicity of nanoparticles used in mitochondria delivery systems^99^. Overall, mitigating immune responses while preserving mitochondrial function is a key focus in the development of safe and effective nanotechnology-based mitochondrial therapies.

### Targeting and delivery efficiency

Nanoparticle-based drug delivery systems aim to transport therapeutic agents directly to target tissues, thereby achieving effective drug concentrations at the target site while minimizing systemic exposure and off-target effects^100^. However, one of the most significant challenges limiting the success of nanoparticles is their ability to reach the therapeutic site in sufficient concentrations while avoiding accumulation in non-target tissues^101^. This targeted delivery is impeded by various physiological barriers, including immune clearance, inefficient cellular uptake, and restricted intracellular diffusion through the cytoplasm^101^. The challenge becomes even more complex when targeting mitochondria, particularly within organs protected by stringent biological barriers like the BBB, as the intricate morphology and compartmentalization of these target tissues further hinder direct mitochondria integration^102^. To overcome these limitations, several strategies have been explored for delivering exogenous mitochondria into cells or tissues, including cell-mediated transfer via TNTs, vesicle-mediated transport, and systemic delivery facilitated by carriers or cell-penetrating peptides^19,67^. While promising, these approaches face technical and biological challenges and must be optimized according to the specific cellular or tissue context^103^. Therefore, to facilitate significant advancements in the field of MTT, it is imperative to elucidate the mechanisms underlying the targeted delivery of mitochondria to damaged tissues.

### Scalability and regulatory hurdles

Nanoparticles offer exceptional versatility, allowing for diverse payloads, compositions, and targeting strategies that can be tailored to a wide range of therapeutic applications^104,105^. However, scaling these systems for clinical use presents significant challenges^106^. The in vitro and in vivo behavior of nanoparticles is strongly influenced by various physicochemical properties, including particle size and size distribution, surface morphology, surface chemistry, charge, adhesion characteristics, and stability^107^. These factors not only affect the biodistribution and therapeutic efficacy of nanoparticles but also their safety profiles^105^. The formulation of nanoparticles typically involves complex procedures such as the use of organic solvents, emulsification, and lyophilization, all of which must be carefully controlled to ensure product consistency and reproducibility^107,108^. A streamlined manufacturing process that is compatible with the intended route of administration is critical to achieving optimal biocompatibility and therapeutic activity^109^. Moreover, for applications involving MTT, the composition of nanoparticles becomes even more critical, as interactions with mitochondrial membranes can impact their structural integrity and functionality^110,111^. An additional critical concern is the economic feasibility of nanoparticle-based therapeutics^112^. The production of clinical-grade nanoparticles often requires high-quality raw materials, specialized equipment, and controlled manufacturing environments that comply with Good Manufacturing Practices^112,113^. These factors significantly elevate production costs and may affect the cost-benefit ratio for widespread clinical implementation^114,115^. Navigating the regulatory process for nanoparticle-based therapies is still a major challenge. Health authorities require large amounts of data on safety, effectiveness, and quality, and it’s often hard to maintain consistency across different production batches due to the complex nature of nanoparticles^116,117^. To bring mitochondrial therapies based on nanotechnology from the lab to clinical use, it’s essential to develop manufacturing methods that are reliable, scalable, and affordable. At the same time, having clear and supportive regulatory guidelines will be key to making these therapies a reality^118^.

### Mechanistic understanding

Host cells can internalize exogenous mitochondria through multiple cellular pathways, facilitating the intercellular exchange of both functional and impaired mitochondria. Studies on mitochondrial tracking have revealed that while some exogenous mitochondria successfully fuse with the host cell’s mitochondrial network^119^, others are directed toward lysosomal degradation^120^. This dual fate underscores the complexity of MTT and the need to better understand the mechanistic determinants that govern mitochondrial uptake, integration, and turnover. To enhance the efficiency of MTT, several advanced delivery strategies have been developed, such as peptide-mediated mitochondria delivery, centrifugation-assisted methods, and the use of EVs for encapsulation and transport^121^. These approaches aim to improve mitochondrial stability, facilitate membrane translocation, and increase cellular uptake, yet their success varies depending on the cellular context and tissue type^122,123^. Given the central role of mitochondrial dysfunction in numerous diseases, there is a pressing need to develop effective mitochondria delivery strategies, particularly for overcoming the BBB, which represents a major physiological obstacle in central nervous system therapeutics. In this context, nanoparticles have emerged as a promising modality for facilitating targeted mitochondria delivery and BBB penetration in neurological disease models^124^. Although progress has been made, our understanding of nanoparticle-mediated MTT—especially when using mitochondria sourced from cells or tissues—remains limited in cell, animal, and clinical studies^125,126^.

### Regulatory and ethical considerations

Addressing ethical concerns is pivotal to the successful clinical translation of biotechnology-mediated MTT. The development and approval of nanoparticle-based therapies present unique challenges due to the complexity of their composition and the diversity of their biomedical applications^127^. Among the most pressing concerns is the potential toxicity of nanoparticles, which can vary significantly depending on their physicochemical properties, including size, charge, composition, and surface characteristics^107^. These factors directly influence biological interactions and may contribute to a range of toxicological outcomes^128^. A key ethical challenge in MTT is the source of the donor mitochondria. In autologous transplantation, mitochondria are isolated from the patient’s own tissues and reintroduced into damaged cells or organs^34^. While this approach minimizes immunogenic and ethical concerns, it still poses biological challenges, such as maintaining mitochondrial viability and function, which require extensive validation in preclinical models^129,130^. A further ethical concern is the potential transmission of foreign mtDNA, especially when using non-autologous sources. If donor mitochondria integrate into germline cells, there is a theoretical risk of passing unintended genetic modifications to future generations, raising significant concerns about heritable genetic alteration without a full understanding of long-term effects. Additionally, the mitochondria isolation and preparation process carries the risk of contamination, which may introduce pathogens or harmful agents into the recipient. This not only undermines treatment efficacy but could also worsen the patient’s condition^131^. As the field of MTT continues to progress, there is a critical need for the development of standardized protocols, certification of clinical settings, and the establishment of robust regulatory frameworks to ensure both safety and therapeutic efficacy. Addressing these multifaceted challenges will require a multidisciplinary approach, integrating expertise from biotechnology, medicine, ethics, and regulatory science.

## Future perspectives in MTT

The field of MTT holds immense potential, with several promising future directions. Below, we synthesize insights from recent literature and present additional perspectives:

### Targeting mitochondria for therapy

Mitochondrial dysfunction, whether hereditary (e.g., Leber’s hereditary optic neuropathy or mitochondrial encephalomyopathy) or acquired, as seen in aging or chronic diseases like diabetes and metabolic syndrome, contributes to cellular senescence and metabolic decline^132–134^. These dysfunctions drive the progression of age- and disease-related conditions. MTT offers a promising approach to restore cellular health and energy balance by directly addressing these dysfunctions. Understanding how transplanted mitochondria integrate into cells, repair or replenish dysfunctional mitochondria, and restore metabolic homeostasis could enable tailored treatments against chronic diseases and aging^135^.

### Enhancing mitochondrial viability

Mitochondrial coating or encapsulation, such as using liposomal carriers, can enhance MTT efficacy and the viability of transferred material. Nanovesicle-mediated transport offers dual benefits: targeting specific tissues and shielding mitochondria from degradation, which is critical since damaged mitochondria can induce proinflammatory pathways, leading to unintended harm^136–138^. Enhancing the recipient cell’s ability to take up transplanted mitochondria is another promising direction. Cells under distress produce “help me” and “save me” signals that attract mitochondria. Manipulating these signals could improve mitochondrial uptake efficiency and specificity in target cells^139^. Increasing the intracellular half-life of transferred mitochondria could reduce the need for multiple infusions. Further research is needed to understand how to increase mitochondrial retention within target cells and improve integration into endogenous mitochondrial networks^140^. Functional augmentation of isolated mitochondria before transplantation is gaining traction, synergizing with advanced biomedical technologies. Enhancements such as boosting antioxidant capacity, modulating metabolic activity, or editing mtDNA can optimize functional benefits while improving resilience post-transfer^141^.

### Optimizing mitochondria delivery

Neurodegenerative disorders like Alzheimer’s and PD present unique challenges due to the BBB. Current delivery methods, such as intrathecal injections, carry risks and limitations. Furthermore, applying isolated mitochondria directly into cerebrospinal fluid may provoke serious immune reactions^142^.

**Fig. 3:**
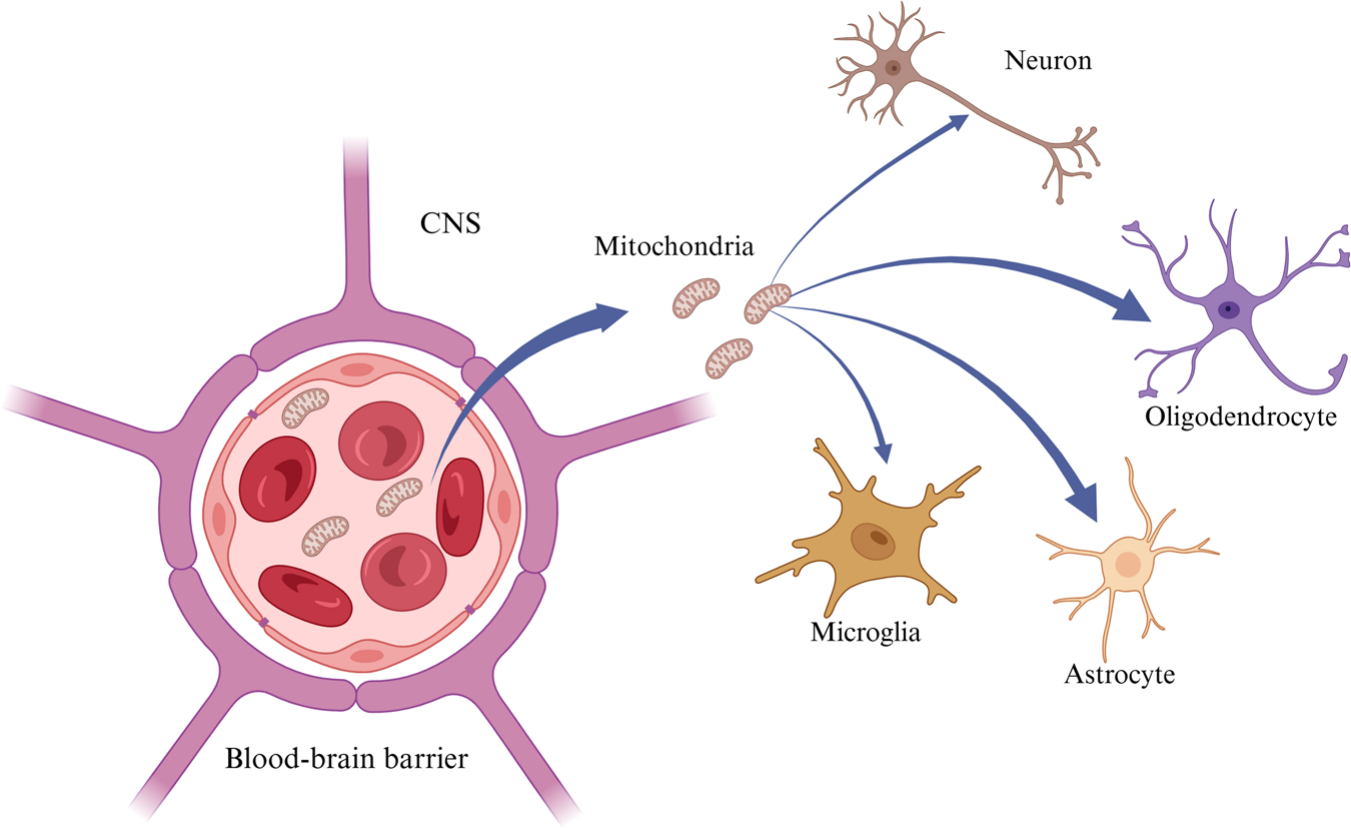
Mitochondria delivery across the blood-brain barrier to central nervous system (CNS) cells. This figure shows that mitochondria reach the brain; they must cross the blood-brain barrier (BBB). Furthermore, once they have reached the brain parenchyma, they must be internalized by neurons, astrocytes, oligodendrocytes, and microglia to perform their function. The entry of mitochondria (red) into the CNS space from the vasculature is shown here, with arrows indicating their potential uptake routes in neurons (brown), oligodendrocytes (purple), astrocytes (light orange), and microglia (gold). The structure of the BBB is shown in stylized purple and pink, and endothelial cells and red blood cells are shown in cross-section. The photograph highlights the clinical potential of MTT in the treatment of CNS disorders, as well as the significant challenge of crossing the BBB—a major obstacle to neurotherapeutic delivery. Created in BioRender. Nuzzo, D. (2025) https://BioRender.com/l33k213.

The administration of mitochondria intravenously via the tail vein into mice afflicted with PD was undertaken to ascertain whether this modality of administration would prove efficacious. Following a 2-h interval, the distribution of mitochondria was evident in multiple tissues, including the brain, liver, kidneys, muscles, and heart. These findings indicate that mitochondrial administration can serve as a preventative measure against the progression of PD^143^. However, there are data to suggest that the efficiency of mitochondria to cross the BBB is reduced and that the pathway of mitochondria across physiological barriers (e.g., BBB) and into the parenchyma remains unclear^144^. Shi et al. have noted that the injected mitochondria are distributed in different organs and that there is no preferential spatial localization. Furthermore, measurement of ATP content has shown that ATP levels are increased in all organs analyzed, with the brain having the lowest levels. Moreover, there is currently a paucity of studies that provide unequivocal evidence on the efficiency of mitochondria crossing the BBB. In addition, once the injected mitochondria have crossed the BBB, they must selectively reach the brain region, and the specific cells (neurons, microglia, astrocytes or oligodendrocytes) are involved in the brain pathology (Fig. 3). To date, various nano-systems such as nanoparticles, liposomes, dendrimers, nanovesicles and nanogels have been studied for drug delivery to the CNS and to specific brain cells^145–149^, but these strategies are still limited for MTT. This suggests that an approach is needed to deliver mitochondria specifically to the brain by crossing the BBB to enhance the effects of brain MTT. Biotechnological approaches, including those mentioned above, may bypass the BBB, deliver mitochondria directly to the brain tissue, and prevent unwanted immune responses, opening new therapeutic avenues for these debilitating conditions^124^.

### Precision and efficiency

Mitochondria isolation is time-intensive, and the product’s limited half-life restricts clinical scalability. Rapid mitochondria delivery could play a crucial role in emergencies like myocardial infarction or cerebral ischemia. Additionally, mitochondrial preparations could address critical challenges in organ transplantation, such as extending the viability window for organs donated after circulatory death. Developing ready-to-use injectable mitochondrial preparations could revolutionize treatment protocols. A standardized vivo culturing of biocompatible isolated mitochondria in large quantities, along with novel short- and long-term storage methods, could ensure a readily available supply for transplantation and significantly expand therapeutic potential^58,150–152^.

### Elucidating mechanisms of MTT

Despite numerous studies demonstrating the benefits of MTT, the exact mechanisms underlying these effects remain poorly understood. Detailed investigations into how transplanted mitochondria interact with host cells, restore organelle function, and integrate into cellular systems are essential to refine therapeutic protocols and improve outcomes. The selection of donor cells, tissues, or species represents a crucial factor. Mitochondria derived from different tissues may possess unique properties influencing their efficacy. Comprehensive documentation and comparative studies of tissue- and species-specific effects are necessary to establish standardized and optimized transplantation practices^129,153^.

### Validation of safety and efficacy in humans

While preclinical studies have shown promise, there is a significant gap in human clinical trials assessing the safety and efficacy of MTT for therapeutic use. Rigorous, large-scale clinical trials are necessary to validate these approaches in human patients, particularly regarding long-term benefits and potential adverse effects^154^.

## Conclusion

The potential of nanotechnology-enhanced MTT to revolutionize the treatment of diseases associated with mitochondrial dysfunction is substantial. The effectiveness of biotechnology in overcoming the current challenges of mitochondrial therapy relies on advancements in the design, targeting, and biocompatibility of nanotechnology driven mitochondria delivery systems. An emerging perspective worth exploring is the co-delivery of functional mitochondria alongside mitochondria-protective agents—such as antioxidants or bioenergetic molecules—through advanced biotechnological platforms. Such a strategy could aim not only to replace dysfunctional mitochondria, but also to protect the transplanted mitochondria in the new cellular environment characterized by mitochondrial dysfunction. Future research should prioritize optimizing these systems to address existing limitations, thereby facilitating their application in regenerative medicine and personalized therapeutic approaches.

## Data Availability

Data sharing does not apply to this article as no new data were created or analyzed in this study.
